# Developing a Low-Cost, Simple-to-Use Electrochemical Sensor for the Detection of Circulating Tumour DNA in Human Fluids

**DOI:** 10.3390/bios10110156

**Published:** 2020-10-28

**Authors:** Bukola Attoye, Chantevy Pou, Ewen Blair, Christopher Rinaldi, Fiona Thomson, Matthew J. Baker, Damion K. Corrigan

**Affiliations:** 1 Department of Biomedical Engineering, University of Strathclyde, 40 George Street, Glasgow G1 1QE, UK; ewen.blair@strath.ac.uk (E.B.); damion.corrigan@strath.ac.uk (D.K.C.); 2 Wolfson Wohl Cancer Research Centre, Institute of Cancer Sciences, University of Glasgow, Glasgow G61 1QH, UK; Chantevy.Pou@glasgow.ac.uk (C.P.); Fiona.Thomson@glasgow.ac.uk (F.T.); 3 Technology and Innovation Centre, Department of Pure and Applied Chemistry, University of Strathclyde, 99 George street, Glasgow G1 1RD, UK; christopher.rinaldi@strath.ac.uk (C.R.); matthew.baker@strath.ac.uk (M.J.B.)

**Keywords:** electrochemical, DNA biosensors, KRAS, liquid biopsy

## Abstract

It is well-known that two major issues, preventing improved outcomes from cancer are late diagnosis and the evolution of drug resistance during chemotherapy, therefore technologies that address these issues can have a transformative effect on healthcare workflows. In this work we present a simple, low-cost DNA biosensor that was developed specifically to detect mutations in a key oncogene (KRAS). The sensor employed was a screen-printed array of carbon electrodes, used to perform parallel measurements of DNA hybridisation. A DNA amplification reaction was developed with primers for mutant and wild type KRAS sequences which amplified target sequences from representative clinical samples to detectable levels in as few as twenty cycles. High levels of sensitivity were demonstrated alongside a clear exemplar of assay specificity by showing the mutant KRAS sequence was detectable against a significant background of wild type DNA following amplification and hybridisation on the sensor surface. The time to result was found to be 3.5 h with considerable potential for optimisation through assay integration. This quick and versatile biosensor has the potential to be deployed in a low-cost, point-of-care test where patients can be screened either for early diagnosis purposes or monitoring of response to therapy.

## 1. Introduction

Cancer is by nature a genetic disease, arising from random mutations and DNA damage which once reaching a certain threshold result in cellular malfunction. At present, detection of cancer is based mainly on clinical presentation of symptoms, which often are vague and non-descript. The time point at which a tumour manifests clinical symptoms often correlates with the later stages of progression (e.g., Phases III and IV) where surgery and therapy are less effective because of e.g., metastasis or tumour cells acquiring a critical mass of mutations [1]. At present, once cancer is suspected, referral to a specialist and the use of imaging techniques such as CT and MRI is the dominant clinical approach to diagnosis [1]. Once a patient is determined to have a tumour, very often a physical biopsy will be taken which involves removing a section of the tumour with a needle. A concept that is gaining increasing traction in cancer diagnosis is the idea of ‘liquid biopsy’ [2]. This is where liquid samples, such as blood [3], urine [4], cerebrospinal fluid etc., are assayed for biomarkers of cancer. The fact that these body fluids are easily accessed by clinicians with minimal damage, risk and pain for the patient means the approach represents an exciting possibility for assaying biomarkers with the aim of identifying early stage tumour formation and measuring response to therapy [5,6]. For identifying early stage tumours via liquid biopsy, a number of potential biomarkers have been proposed, but, two major classes of markers of particular importance are circulating tumour cells (CTCs) and circulating tumour DNA (ctDNA) [7]. Both markers have high diagnostic and predictive capability with each having advantages and disadvantages. Assaying for CTCs means having to concentrate low numbers of cells (e.g., 10 cells/1 mL blood) for detection whereas assaying for ctDNA means identifying a lower number of copies of the mutant sequence against a very high background of wild type sequences. In selecting the approach reported here, ctDNA was chosen as the biomarker because its detection could potentially be coupled to a DNA amplification reaction and because it allowed the possibility of developing a multiplexed panel of DNA sequences on a single chip, meaning that commonly mutated genes could all be identified in parallel (e.g.,KRAS, TP53, BRCA1&2, IDH-1, etc.,). This approach of developing biomarker ‘panels’ is thought to be one of the key advantages of the approach [8].

Electrochemical DNA biosensors represent an exciting approach to the fast, low-cost detection of clinically important biomarkers [9,10] at the point of care [11]. A range of electrode materials and electrochemical measurement approaches have been employed for sensitive measurements (cyclic voltammetry [12], differential pulse voltammetry [13], square wave voltammetry [14] and electrochemical impedance spectroscopy [15]). The potential of electrochemical biosensors, once matured as a technology to provide efficient clinical workflows is immense. The topic of liquid biopsies is one such area where innovations in biomarker detection will enhance clinical outcomes for patients [16]. Electrochemical systems for ctDNA detection or cancer biomarker detection have most often employed the ‘three electrode system’ where the working electrode is functionalised with a nucleic acid probe sequence and then interrogated using some measurement techniques to determine recognition of the target molecule [17]. In addition to the evaluation of potential measurement and signal amplification schemes, the importance of the underlying electrode has also been investigated through the use of e.g., nanostructured gold electrodes [18]. What these advances have so far reported are complex assays involving the use of modified oligonucleotides [19], formation of branched structures upon DNA recognition [20], molecular switches [21], nanostructured electrode modifications [18], etc., therefore ultimately having a limited chance of manufacture and clinical uptake. The current state-of-the-art in terms of general nucleic acid liquid biopsies [22] and electrochemical detection of ctDNA biomarkers [23] have been well-reviewed recently.

This work presents a KRAS G12D DNA oligonucleotide probe-modified sensor which when coupled to a PCR reaction detects mutated amplicons from a significant background of human DNA and in particular KRAS wild type sequences (KRAS G12D mutation occurs at position 35 on chromosome 12 changing glycine to aspartic acid in the protein structure). The advantage of our approach is its simplicity. A straightforward covalent attachment is used to couple a simple amine-modified DNA sequence (e.g., no tethered labels or redox intercalators) to a simple screen-printed carbon electrode which is then interrogated using cyclic voltammetry (CV) to reveal DNA hybridisation and thus mutation detection in potassium ferri-ferrocyanide solution. Because of the simplicity of the approach presented herein and the choice of steps employed in the assay, the system can be very easily automated and integrated into a final device capable of fast and seamless clinical measurements. Since the assay has been deliberately engineered to be simple, low-cost and employs well-established methodologies such as PCR, the hope is that it will lead to faster clinical adoption.

## 2. Materials and Methods

### 2.1. Reagents

Droplet digital PCR (ddPCR) assays, Supermix for probes, DG8TM cartridges and Droplet Generation Oil were obtained from BioRad Laboratories Ltd., UK. Deionised water, sodium chloride, phosphate buffered saline (PBS), sodium nitrate, 4-aminobenzoic acid, hydrochloric acid, ethanolamine, 2-(*N*-morpholino) ethanesulfonic acid (MES), 1-ethyl-3-(3-dimethylaminopropyl) carbodiimide hydrochloride (EDC), N-hydroxysuccinimide (NHS), potassium ferricyanide, potassium chloride and potassium ferrocyanide were all purchased from Sigma-Aldrich (Dorset, UK). 250 units HotStarTaq Plus and dNTP Mix, PCR Grade (200 μL) were purchased from Qiagen, (Manchester, UK). Phusion direct PCR kit was purchased from thermo scientific, (Renfrew, UK).

### 2.2. Electrochemical Setup

**Figure 1:**
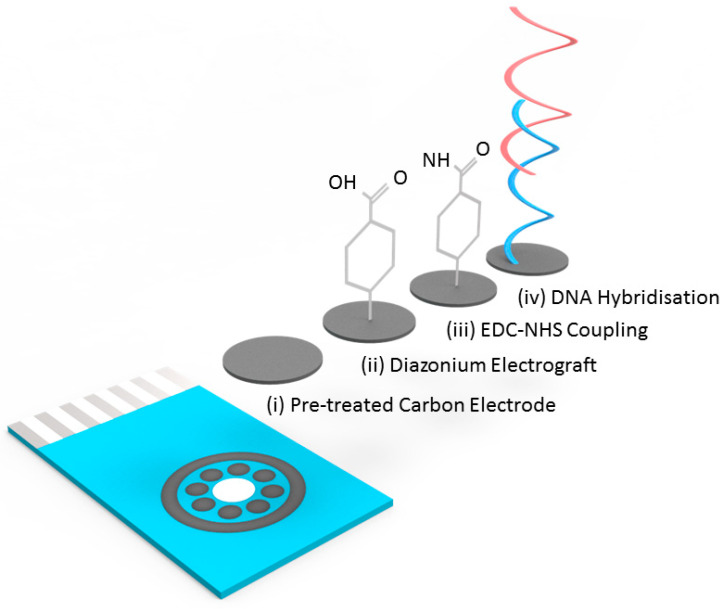
Image of screen printed electrode array employed (8 × working electrodes with common Ag reference and carbon counter electrodes along with schematic showing DNA functionalisation and DNA target binding.

Screen printed multi carbon electrodes (DRP 8W110) as shown in Figure 1 below, were obtained from DropSens (Oviedo, Spain) with chip dimensions of 50 × 27 × 1 mm (L × W × D). The chip contained eight carbon working electrodes with diameters of 2.95 mm with a carbon counter and silver reference electrode. The screen printed fabrication process is specified by the manufacturers.

### 2.3. Electrochemical Measurements and Surface Functionalisation

DNA hybridisation experiments were performed using a covalently attached layer of single-stranded DNA probes. The surface functionalisation protocol is illustrated in Figure 1. To prepare the surface of the carbon electrodes for DNA probe attachment it was necessary to first use a surface pretreatment by applying 1.4 V for 1 min in 0.5 M acetate buffer solution (ABS) containing 20 mM NaCl 8 (pH 4.8). Next, 2 mM NaNO_2_ solution with 2 mM 4-aminobenzoic acid was prepared in 0.5 M HCl and stirred for about 5 min at room temperature to produce a diazonium compound. The activated diazonium solution was then scanned using cyclic voltammetry from +0.4 to −0.6 V at a scan rate of 100 mV/s followed by a wash with DI water. The resulting 4-carboxyphenyl (AP) film was activated on the electrode surface with 100 mM EDC and 20 mM NHS in 100 mM MES buffer (pH 5.0) for 60 min to form an ester that allowed for efficient conjugation to the amine modified ssDNA probe (Figure 1). Unless specifically stated, all the reported steps and measurements were carried out at room temperature.

### 2.4. Genomic DNA Sample Preparation, DNA Probe Design and Sample Amplification

For assay development, copies of the KRAS pG12D mutant and wild type DNA were amplified from genomic DNA (gDNA) isolated from SK-UT-1 cells. Levels of both mutant and wild type DNA were determined using ddPCR assays (KRA p.G12D c.35G>A, assay ID dHsaCP000001and KRAS WT for p.G12D c.35G>A, assay ID dHsaCP2000002) in combination with a QX200TM Droplet DigitalTM PCR system (Biorad Laboratories Ltd., Hertfordshire, UK) following the manufacturer’s instructions. Briefly, 5–10 ng of gDNA isolated from SK-UT-1 cells- was mixed with ddPCR Supermix for probes (N0 dUTP) and florescein amidite (FAM)-labelled KRAS p.G12D primers/probe and hexachloro-fluorescein 9 (HEX)-labelled KRAS WT primers/probes, in the presence of restriction enzyme and in a volume of 20 μL. Reaction samples were loaded onto a DG8TM cartridge with 70 μL of Droplet Generation Oil for Probes according to the Droplet Generator Instruction Manual (Biorad Laboratories Ltd., UK). PCR cycling conditions for the generated droplets were as follows: initial enzyme activation at 95 °C for 10 min, followed by 40 cycles of denaturation at 94 °C for 30 s and annealing/extension at 55 °C for 1 min, after which ended with a final enzyme deactivation at 98 °C for 10 min. Data acquisition after thermal cycling, was performed using the QX200 Droplet Reader and the QuantaSoft Software (Biorad Laboratories Ltd., Hertfordshire, UK).

The design of PCR primers and probes used in this study was based on the published sequence of KRAS pG12D under accession number NC_000012.12 (NCBI, 2019). Amine-modified synthetic oligonucleotides (KRAS G12D) designed as shown in Table 1 below with a concentration of 200 μM were obtained from Sigma-Aldrich, UK and stored at −80 °C prior to aliquoting for use as probes. A wild type probe (without the single base mutation) was also designed for use as a negative control. DNA probe stocks were diluted to a concentration of 20 μM in 0.1 × PBS prior to immobilisation. Primer-BLAST software was used to design the PCR primers employed in this study. The forward primers had a GC content of 55% while the reverse primer had a GC content of 39.13% with an estimated product length of 88 with low self-complementarity.

For standard PCR amplification, samples of extracted wild type and KRAS G12D mutated DNA were amplified using the HotStar Taq plus DNA polymerase with 10 ×P CR Buffer, dNTP mix, Q-solution and primer solutions (Qiagen, UK). Template DNA (≤1 μg/100 μL reaction) was added to individual PCR tubes containing the pre-prepared reaction mix and the thermal cycler was programmed to start with an initial heat-activation step at 95 °C for 5 min. Temperatures specifications for denaturing, annealing and extending were set at 1 min for 94 °C, 65 °C and 72 °C respectively. A final extension for 10 min at 72 °C was set and PCR conditions were systematically varied by employing cycle numbers of 20, 25 and 30. PCR amplification of wild type and KRAS G12D samples was performed using the minipcr thermal cycler and amplicons were characterised using the minipcr blueGel Analyser with 1.5% agarose gel electrophoresis at 140 V for 40 min (Amplyus, 2017).

The phusion blood direct PCR Kit protocol and reaction setup guide outlined by Thermo Scientific, UK was used to amplify the mutants in the total isolated DNA spiked into human plasma. Phusion blood II DNA polymerase (1 µL), 2 × PCR Buffer (25 µL), 50 mM EDTA (2.5 µL), 50 mM MgCl_2_ solution (1.5 µL) and 100% DMSO (2.5µL) were all included in the reaction mix and dispensed into appropriate PCR tubes. Total of 1 μL template DNA, 5 µL forward and reverse primers and 10 μL ultrapure water were added to the master PCR tube containing the reaction mix and the thermal cycler was programmed to start with an initial heat-activation step at 98 °C for 300 s. Temperatures specifications for denaturing, annealing and extending were set at 90 s for 94 °C, 65 °C and 72 °C respectively. A final extension for 60 s at 72 °C was set and PCR conditions was set for 37 cycles. PCR was performed using the minipcr thermal cycler and PCR amplification products were confirmed using minipcr blueGel at 1.5% agarose gel electrophoresis at 140 V for 40 min [24].

## 3. Results and Discussion

### 3.1. Assay Workflow and DNA Amplification

**Figure 2:**
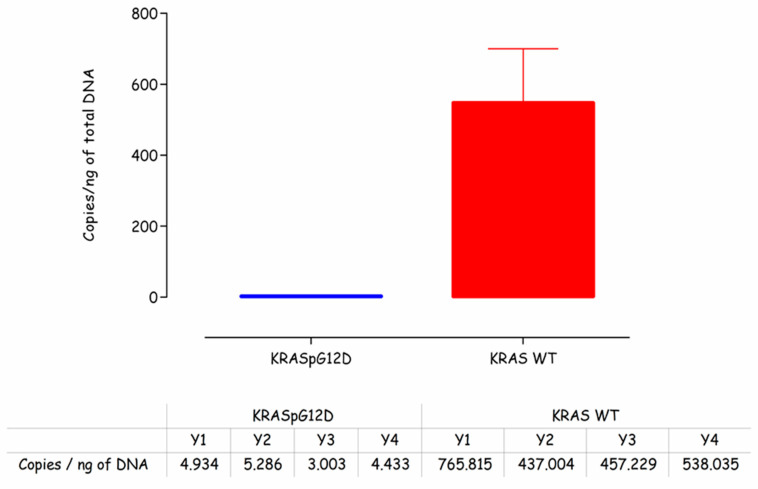
Copies of KRASG12D mutant and KRAS wild type detected in total DNA isolated from SK-UT-1 cells using ddPCR (BioRad QX200).

Prior to carrying out any work on the electrochemical detection of the KRAS G12D mutation, it was necessary to first confirm the presence of mutated DNA in the representative sample. These samples were clinically derived from cancer patients and were employed for assay development to give confidence that the sensor indeed could work with clinical samples. This was done using droplet digital PCR on DNA samples isolated from patients. From Figure 2, it can be seen that the levels of mutated KRAS G12D gDNA were low (~4 copies/ng of total DNA) compared to the wild type KRAS (~550 copies/ng of total DNA). This finding was reassuring, i.e., that KRAS G12D mutations were present in the assay samples but underlined the analytical challenge of amplifying the mutation against a high background of wild type KRAS DNA sequences and also the high genomic DNA background more generally.

In order to couple DNA amplification directly to the electrochemical assay, it was necessary to develop a standard PCR reaction which we chose to run in a portable PCR machine manufactured by ‘minipcr’ (Cambridge, MA, USA) to closely mimic a system which could be deployed in the clinic. To produce a working PCR reaction, primer sets were designed which would specifically amplify the KRAS G12D-mutated sequence or the wild type KRAS sequence. The final sequences are shown in Table 1 (see Materials and Methods section) with the reaction being designed to utilise a common reverse primer. The ability of the primer sets to successfully amplify both the KRAS G12D mutant sequence and the KRAS wild type sequence was confirmed by gel electrophoresis and through measurement on a Qubit fluorometer. These results confirmed our ability to amplify and quantify mutated and wild type sequences from the KRAS G12D sample for electrochemical detection.

Having established both the presence of low copy numbers of KRAS G12D mutations in the sample and an amplification reaction which could produce either mutated or wild type amplicon depending on primer choice, the next stage involved successfully establishing an electrochemical measurement to verify the presence of either wild type or mutant DNA.

### 3.2. Initial Assay Development

Carbon has many advantages that led to its selection as the electrode material in this work. These include: low-cost production, the existence of many established manufacturing process for carbon SPE production, high resistance to biofouling and well established surface functionalisation chemistries. The existence of a multi carbon electrode chip (8 × working electrodes) already commercially available from DropSens (Oviedo, Spain) meant that there was an established format available for initial assay development. With a carbon electrode in hand, it was necessary to implement a surface functionalisation chemistry and to this end functionalisation with a diazonium compound followed by NHS-EDC coupling of amine-tagged DNA, the electrode surface was implemented. This is a well-established and robust methodology for biofunctionalising carbon surfaces for electrochemical readout of bio-recognition events [25,26]. The chip itself and a flow diagram of the functionalisation protocol are shown in Figure 2, full details of the electrode biofunctionalisation process are given in the materials and methods section. Specific probe sequences for the G12D mutant and wild type KRAS sequences were designed, specifically for optimal discrimination of single base-pair changes. Previous work on electrochemical detection of single base-pair changes has demonstrated enhanced discrimination and optimal signal levels when the mutation is placed close to the centre of the probe sequence [26].

**Figure 3:**
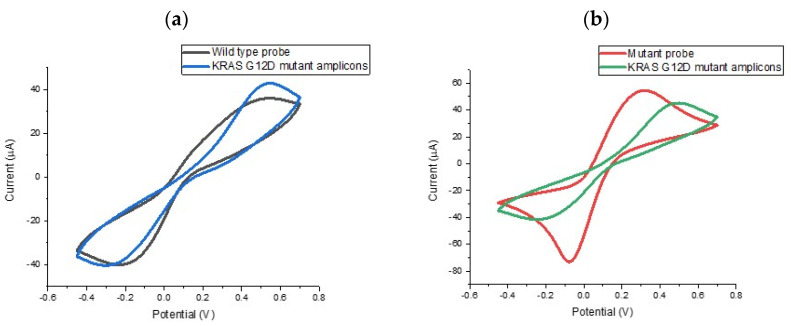
CV results showing hybridisation selectivity between a (**a**) wild type probe (**b**) mutant probe and a representative sample containing PCR amplified KRAS G12D mutant allele with a total KRASG12D ssDNA concentration of 0.854 ng/µL.

In order to evaluate the specificity of the assay, we explored the ability of the probe-modified electrodes to discriminate between G12D mutant and wild type KRAS sequences. To investigate specificity levels and gain an initial impression of assay sensitivity, a series of electrodes were functionalised with KRAS G12D mutant and wild type probe. The results of these experiments are summarised in Figure 3, which shows the percentage change in the reductive peak current following target hybridisation. For macroscale electrodes functionalised with biological molecules such as DNA or antibodies the expectation is that cyclic voltametric peak currents will reduce upon target hybridisation. For impedimetric measurements, it is expected that the charge transfer-resistance (R_CT_) will increase. This is because the presence of greater amounts of DNA at the electrode surface (brought about by target hybridisation) will lead to both electrostatic repulsion of the redox mediator and steric hindrance around the interface, causing a reduction in mass transport limited currents, manifesting in reduced peak heights in voltammetry and increased charge transfer-resistance in EIS. It has been observed that these effects can be reversed when micro or nanoscale electrodes are employed [10,27], but for this study, the electrodes were comfortably employed on the macro scale (diameter = 2.95 mm). Therefore, we would expect a reduction in peak current when specific DNA hybridisation took place and this was found to be the case. When the wild type sequence was hybridised with the mutant PCR product, reductions in the peak current were not observed indicating no significant hybridisation. These findings were highly satisfying, i.e., surface tethered KRAS G12D mutant probe sequence could in fact discriminate between the mutant and wild type sequences from the PCR product). This represented a double specificity for the PCR-based assay because the primer design had already been shown to specifically amplify the mutated sequence so coupling the specificity of the electrochemical probe sequence meant that the assay would be able to specifically identify mutant amplicons from the sample. Having established the specificity of the assay and the nature of the electrochemical change, the next step involved verifying the sensitivity of the assay and dose response effects for the KRAS G12D mutant PCR product.

**Figure 4:**
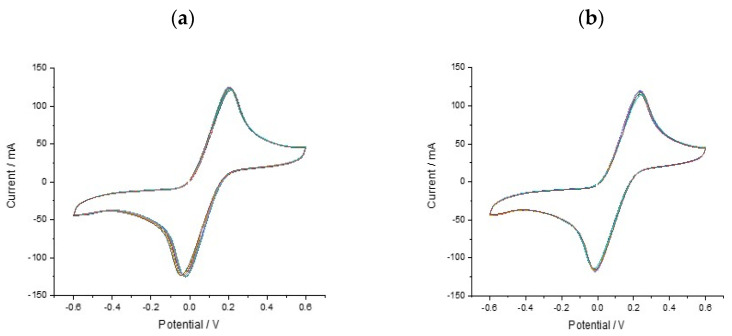
CV results showing repeated cycling of (**a**) KRAS G12D probe functionalised electrodes (**b**) KRAS G12D amplicons over 8-chip multielectrodes using the same current and voltage inputs.

With a functionalised electrode surface, it became necessary to evaluate the reproducibility and stability of the multi-carbon electrode chip. The repeatability of the proposed sensor was evaluated by performing 8 replicates using the same SPCE sensor and ferricyanide solution (Figure 4). The data (*n* = 8) show less variation and more consistency in the signal change. This shows good reproducibility of the SPCE sensor when functionalised with DNA and means that the measurements are easier to interpret. All ferri-ferrocyanide solutions were cycled on the 8-chip multielectrodes (un and DNA functionalised) × 3 times using CV scans from +0.4 to −0.6 V at a scan rate of 100 mV/s with current ranges between 10 nA and 10 mA.

### 3.3. Assay Optimisation

**Figure 5:**
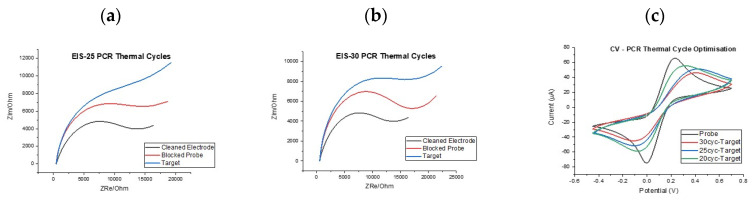
EIS responses for clean, probe modified and target bound electrodes following 25 PCR cycles (**a**) and 30 PCR cycles (**b**,**c**) CV results showing the correlation between oxidative and reductive peak currents and PCR cycle number.

With the basis of the assay established, i.e., PCR primer specificity, ssDNA probe specificity and electrochemical signal changes of the correct direction and magnitude, it was important to investigate whether dose-response effects could be established and whether an optimal number of PCR cycles could be identified to reduce sample to result time. First of all, it was decided to investigate the effect of the number of PCR cycles employed for amplification on the electrochemical signal change post hybridisation. For this work, both cyclic voltammetry (CV) and electrochemical impedance (EIS) measurements were performed in order to determine whether consistent behaviour was observed using the two techniques. Figure 5 shows two example of impedance results showing the changing signal levels for electrodes incubated with PCR product generated from 25 and 30 cycle reactions. It can be seen that the increase in the charge transfer resistance (R_CT_) semi-circle and equivalent circuit fitting using the well-established Randles’ equivalent circuit were expected. For clarity, the expected trend was observed in that R_CT_ increased from clean electrode, to ssDNA mutant probe-modified surface, to target hybridisation with all steps providing clear indication of increased blocking of the electrode surface. Furthermore, the increase in R_CT_ from the probe modification to the target hybridisation step was greater for the 30-cycle PCR reaction when compared to the 25-cycle reaction hinting at a dose response effect, since the 30 cycle reaction would have produced significantly more PCR product (see Figure S1 for R_CT_ values). This was an encouraging finding but repeated functionalisation with ssDNA probe and target hybridisation across multiple sensor chips led to a wide range of impedance responses, sometimes with the response having a profile that was difficult to fit to any established equivalent circuit. Because of this high variation in the impedance response it was decided to focus on voltammetry measurements because these showed high consistency across sensor chips with variation in signal profile significantly reduced. Figure 5c shows an example response and how both the reductive and oxidative currents reduced in a systematic fashion as PCR cycle number increased. This finding was encouraging because it demonstrated clear discrimination between the baseline response and the response following incubation with a 20 PCR cycle KRAS G12D amplicon meaning detection was possible in as few as 20 PCR cycles.

### 3.4. DNA Target Controls

**Figure 6:**
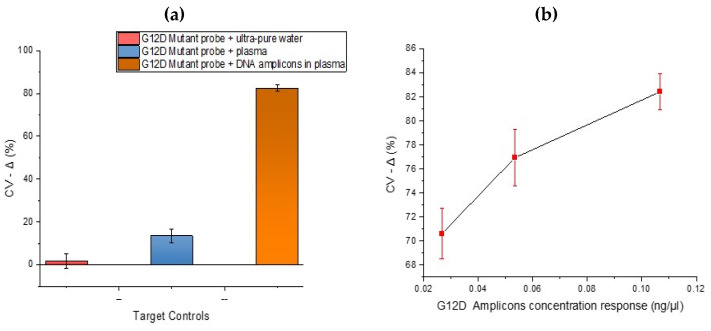
(**a**) CV results showing DNA hybridisation target controls using (i) G12D mutant probe in ultrapure water (ii) G12D mutant probe in human plasma (iii) G12D mutant probe in representative sample containing KRAS G12D mutant amplicons in plasma (**b**) CV results (% signal change) showing G12D amplicons concentration dose response.

After analysing the specificity of the developed DNA sensor, it was necessary to investigate the response effects using assay negative controls. In Figure 6 below, samples investigated ‘as Target hybridisation negative controls’ were ultrapure water and human plasma. In these plots, low signal change arise from small reduction in oxidative peak current post hybridisation. The ultrapure water and plasma showed low signal changes upon hybridisation using a mutant probe. This result is comparable with the hybridisation selectivity performed in Figure 3 with KRAS DNA amplicons. The wide error bars from the plasma immobilisation are assumed to be due to inter-electrode variability and different fragment lengths of non-specific DNA in plasma (especially wild type KRAS) binding to the mutant probe. The hybridisation between the G12D mutant probe and plasma highlights the importance of mutant amplification and is very important as the concentration of circulating free DNA released by tumour cells is usually in proportion to the stage of cancer [28]. After exploring DNA hybridisation controls, it was necessary to investigate the concentration response effects to confirm the potential for quantitation. In Figure 6b, the concentration response for a 25-cycle PCR product diluted to different concentrations was explored in order to gain an impression of potential detection limits. The sample with the lowest concentration (0.027 ng/µL) showed the lowest percentage signal change upon hybridisation. As circulating nucleic acids are present in blood at ng/mL levels, which based on the fragment length is analogous to a picomolar concentration, a minimum of femtomolar sensitivity will be beneficial for detection of tumour-specific sequences without amplification [16]. Whilst a reduction in signal was observed after incubating both the mutant probes in samples of plasma spiked with DNA from the representative patient samples, it is important to note that signal reductions were not as large i.e., a whole mutant amplification reaction when incubated on the sensor brought about much larger decreases in signal, e.g., >80%. This is important because at present, whilst dose response is achievable, the assay was run as a ‘threshold’ test with interrogation of a whole amplification reaction giving rise to clear, detectable changes (above negatives) when the KRAS G12D mutation was present.

The workflow implemented in this assay had a total time to result of 3.5 h. As things currently stand this is very competitive with established clinical approaches to characterising tumour samples e.g., standard pathology techniques. In theory, a tumour can be typed by established techniques in 2–3 days, in practice, using the example of the UK NHS it can take up to 9 weeks in total for results to be confirmed. Therefore, this approach is well positioned to offer substantial cost and time savings to healthcare systems. In addition, the workflow reported is entirely un-optimised with considerable potential for producing a result more quickly through use of isothermal DNA amplification techniques, integration with sample handling technologies and full optimisation of the electrodes and electrochemical measurements procedure, e.g., use of pulsed voltametric techniques in order to heighten sensitivity.

By combining a PCR reaction designed to amplify KRAS mutations from clinical samples it has been possible to demonstrate the detection of G12D mutations by electrochemical means. The electrochemical detection system employed was deliberately designed to be simple and utilise low cost materials in order to result in a cost-effective test once commercialised. The generic nature of the techniques involved, i.e., surface attachment chemistry, PCR, ssDNA probes and electrochemical detection mean that the assay can be very easily enlarged by changing primer and probe sequences to match the mutations required. For example, many common liquid biopsy markers: KRAS, TP53, BRCA1&2 can be detected via this method to give a panel of markers on the electrode array. This will lead to the ability to screen for common cancerous mutations in fast times and at low cost in the clinical environment. In addition to this, the approach can be used to monitor treatment response and give early warning of relapse.

## 4. Conclusions

It was possible to design a PCR reaction capable of amplifying either mutant KRAS G12D or wild type KRAS through primer choice from patient samples. In parallel, an electrochemical detection scheme involving cyclic voltammetry and carbon screen printed electrodes was developed and shown through a series of comparative measurements to be sensitive and specific for the KRAS G12D mutation. Electrochemical impedance spectroscopy measurements were halted because of the inconsistency of the response and difficulties with equivalent circuit fitting. Cyclic voltammetry measurements provided the desired response and showed detection was possible from samples containing 4 copies/ng total DNA in as few as 20 PCR cycles. In addition, the response was found to be consistent, i.e., large signal decreases being evident upon amplification of the mutant allele, offering promise of quantitation of mutant sequences from clinical samples. Sources of potential signal contamination were examined and found to not contribute significantly to the analytical response, giving confidence that signal changes were not due to other binding effects from the sample solutions. These results raise the prospect of simple, rapid, cost effective measurement of nucleic acid tumour markers from blood and other body fluids.

## Figures and Tables

**Table 1:** List of DNA sequences employed in this study.

KRAS Probe and Primer Sequences	
23 bases Wild type Probe	AGTTGGAGCTGGTGGCGTAGGCA
23 bases Mutant Probe	AGTTGGAGCTGATGGCGTAGGCA
Forward Primer (Wild type)	TGTGGTAGTTGGAGCTGGTG
Forward Primer (Mutant)	TGTGGTAGTTGGAGCTGATG
Reverse Primer	TTGTGGACGAATATGATCCAACA

## Supplementary Materials

The following are available online at https://www.mdpi.com/2079-6374/10/11/156/s1, Figure S1: Equivalent circuit models of the impedance spectra recorded for SPCE (a) 25 PCR thermal cycles pre-hybridisation (b) 25 PCR thermal cycles post-hybridisation (c) 30 PCR thermal cycles pre-hybridisation (d) 30 PCR thermal cycles post-hybridisation.
