# Supplementary material for: Developing a Low-Cost, Simple-to-Use Electrochemical Sensor for the Detection of Circulating Tumour DNA in Human Fluids
Source: Biosensors (Basel). 2020 Oct 28;10(11):156. doi: 10.3390/bios10110156 (PMC7692145; doi:10.3390/bios10110156)
Supplement: Supplementary file 1 [file biosensors-10-00156-s001.pdf]

biosensors

MDPI

Supplementary Information

Developing a low cost, simple to use electrochemical sensor for the detection of circulating tumour DNA in human fluids

Bukola Attoye [1,*], Chantevy Pou [2], Ewen Blair [1], Christopher Rinaldi [3], Fiona Thomson [2], Matthew J. Baker [3] and Damion K. Corrigan [1]

1 Department of Biomedical Engineering, University of Strathclyde, 40 George Street, Glasgow G1 1QE, UK; ewen.blair@strath.ac.uk (E.B.); damion.corrigan@strath.ac.uk (D.K.C.)
2 Wolfson Wohl Cancer Research Centre, Institute of Cancer Sciences, University of Glasgow, Glasgow G61 1QH, UK; Chantevy.Pou@glasgow.ac.uk (C.P.); Fiona.Thomson@glasgow.ac.uk (F.T.)
3 Technology and Innovation Centre, Department of Pure and Applied Chemistry, University of Strathclyde, 99 George street, Glasgow G1 1RD, UK; christopher.rinaldi@strath.ac.uk (C.R.); matthew.baker@strath.ac.uk (M.J.B.)
* Correspondence: bukola.omolaiye@strath.ac.uk

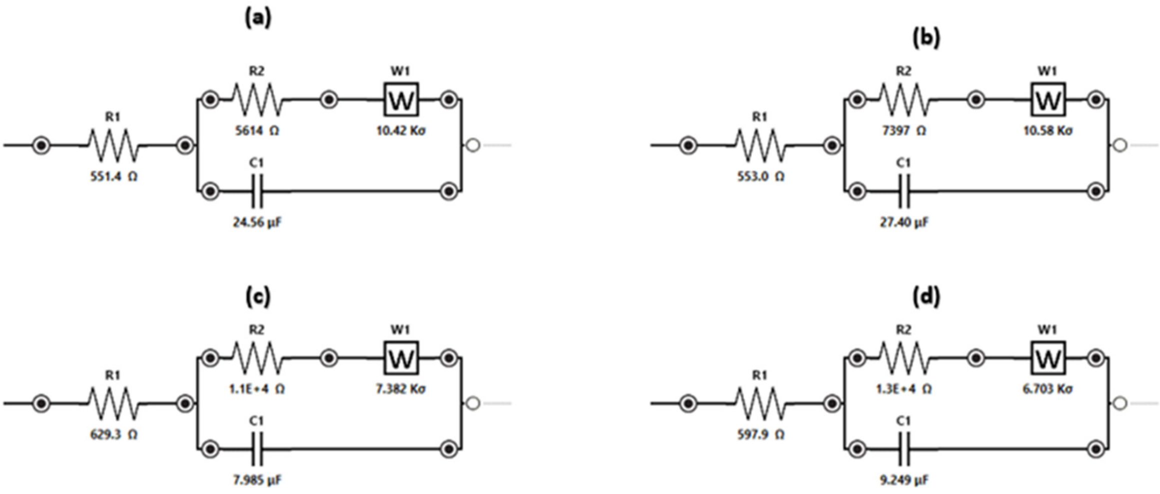

Figure S1. Equivalent circuit models of the impedance spectra recorded for SPCE (a) 25 PCR thermal cycles pre-hybridisation (b) 25 PCR thermal cycles post-hybridisation (c) 30 PCR thermal cycles pre-hybridisation (d) 30 PCR thermal cycles post-hybridisation.
